# Cognitive Biases Questionnaire for Psychosis (CBQp): Spanish Validation and Relationship With Cognitive Insight in Psychotic Patients

**DOI:** 10.3389/fpsyt.2020.596625

**Published:** 2021-02-18

**Authors:** Lia Corral, Javier Labad, Susana Ochoa, Angel Cabezas, Gerard Muntané, Joaquín Valero, Vanessa Sanchez-Gistau, Maribel Ahuir, David Gallardo-Pujol, Josep María Crosas, Diego Palao, Elisabet Vilella, Alfonso Gutierrez-Zotes

**Affiliations:** ^1^Institut Pere Mata, Hospital Universitari Institut Pere Mata of Reus, Reus, Spain; ^2^Centro de Investigación Biomédica en Red de Salud Mental (CIBERSAM), Madrid, Spain; ^3^Institut d'Investigació Sanitària Pere Virgili (IISPV), Tarragona, Spain; ^4^University of Rovira i Virgili, Tarragona, Spain; ^5^Autonomous University of Barcelona, Barcelona, Spain; ^6^Instituto de Investigación e Innovación Parc Taulí (I3PT), Barcelona, Spain; ^7^Sant Joan de Déu Research Institute (IRSJD), Esplugues de Llobregat, Spain; ^8^Parc Sanitari Sant Joan de Déu, Sant Boi de Llobregat, Spain; ^9^Faculty of Psychology, University of Barcelona, Barcelona, Spain; ^10^Institute of Neurosciences, University of Barcelona, Barcelona, Spain

**Keywords:** cognitive bias, psychosis, delusion, cognitive insight, self-certainty

## Abstract

**Introduction:** Cognitive biases are key factors in the development and persistence of delusions in psychosis. The Cognitive Biases Questionnaire for Psychosis (CBQp) is a new self-reported questionnaire of 30 relevant situations to evaluate five types of cognitive biases in psychosis. In the context of the validation of the Spanish version of the CBQp, our objectives were to (1) analyze the factorial structure of the questionnaire with a confirmatory factor analysis (CFA), (2) relate cognitive biases with a widely used scale in the field of delusion cognitive therapies for assessing metacognition, specifically, Beck's Cognitive Insight Scale (BCIS) (1), and, finally, (3) associate cognitive biases with delusional experiences, evaluated with the Peters Delusions Inventory (PDI) (2).

**Materials and Methods:** An authorized Spanish version of the CBQp, by a translation and back-translation procedure, was obtained. A sample of 171 patients with different diagnoses of psychoses was included. A CFA was used to test three different construct models. Associations between CBQp biases, the BCIS, and the PDI were made by correlation and mean differences. Comparisons of the CBQp scores between a control group and patients with psychosis were analyzed.

**Results:** The CFA showed comparative fit index (CFI) values of 0.94 and 0.95 for the models with one, two, and five factors, with root mean square error of approximation values of 0.031 and 0.029. The CBQp reliability was 0.87. Associations between cognitive biases, self-certainty, and cognitive insight subscales of the BCIS were found. Similarly, associations between total punctuation, conviction, distress, and concern subscales of the PDI were also found. When compared with the group of healthy subjects, patients with psychoses scored significantly higher in several cognitive biases.

**Conclusion:** Given the correlation between biases, a one-factor model might be more appropriate to explain the scale's underlying construct. Biases were associated with a greater frequency of delusions, distress, conviction, and concern as well as worse cognitive insight in patients with psychosis.

## Introduction

Cognitive biases are involved in the development and persistence of delusions (3–5). They occupy a central place in recent biopsychosocial models of psychosis (4, 6–8), both in terms of the content of thought (9, 10) and in the processes of reasoning and meta-cognition (4, 11). Cognitive and training therapies in metacognition base their active principle of intervention regarding delusions on the modification of cognitive biases (12). Metacognitive training decreases cognitive biases and improves positive symptomatology in psychosis (13–19).

People with delusions tend to show different reasoning biases. The most researched biases are jumping to conclusions (JTC) (3, 4, 20), attributional biases (21, 22), inflexibility in beliefs, and theory of mind deficits (ToM) (3, 23).

The JTC bias in patients implies that they tend to consider fewer data to arrive at a decision than healthy controls (23, 24), which has been observed in between one-third and two-thirds of subjects with delusions (4, 25–28).

JTC has also been found in healthy subjects with delusion-like experiences (28), subjects at high risk of suffering from psychosis (29), subjects with active psychotic symptoms at the time of evaluation (4), and in a more attenuated manner in psychotic patients' relatives (28, 30). Colbert and Peters (31) and Ross et al. (32) also found a significant presence of JTC in healthy people prone to delusions.

The results of a meta-analysis imply that JTC supposes an increase in the probability of the appearance of delusions in psychosis (33). This bias was found in people with psychosis who tended to look for less evidence when making decisions and who used more “extreme responses” when compared with both healthy subjects and subjects with other mental illnesses different from psychosis. The meta-analysis likewise concludes that there is an inverse relationship between data search and the severity of delusions.

Another meta-analysis implies that JTC would not be a transdiagnostic phenomenon of psychosis (34). It is specifically associated with delusions rather than with the diagnosis of schizophrenia and may contribute to its severity (35). Therefore, JTC is a stable feature that increases the vulnerability to the development of delusions and can predict the changes over time (36).

On the other hand, regarding attribution biases, some studies show evidence of an externalization–personalization bias for negative events in people with persecution delusions in comparison with healthy controls (37–40). Patients with symptoms of paranoia have a greater personalization bias for negative events than patients without these symptoms, and this bias is still evident in remission phases (41).

A review article concludes that deficits in ToM may be characteristic of schizophrenia because, despite being found in patients with delusions, they seem to be more strongly associated with negative and disorganized symptoms than specifically with delusions (23).

Although emotional-type biases have been associated with psychotic thinking (8, 42), few studies have linked Beck's described biases for emotional disorders with psychotic symptoms. Nonetheless, biases such as dichotomous thinking, emotional reasoning, and catastrophising have been associated with delusional symptomatology (4, 8).

Despite associations of internal emotional states with delusions, until the appearance of the Cognitive Biases Questionnaire for Psychosis (CBQp) (18), there was no scale to specifically measure Beck's biases in patients with psychosis.

The CBQp (18) was developed to assess cognitive biases in psychosis. It is based on the Blackburn Cognitive Styles Test (43), which was designed to assess common cognitive distortions in depression and amended to provide appropriate scenarios for psychotic patients. For the validation of the CBQp, the structure, validity, and reliability of the scale was analyzed in a group of subjects with psychosis. The CBQp scores were compared with those of depressed subjects and healthy controls. The results showed adequate internal consistency and test–retest reliability. The items of the scale had a bifactorial structure, implying that the five cognitive biases would not be independent. This result suggests the possibility that the CBQp evaluates a general thinking bias rather than different cognitive errors. The scores obtained in the anomalous perception (AP) and threatening events (TE) themes could be independently used.

Subjects with psychosis and those with depression obtained higher total CBQp scores than healthy controls (18). Subjects with active psychotic symptoms at the time of the evaluation obtained higher scores than the asymptomatic subjects, showing modest associations between the CBQp scores, and the severity of symptoms. The scores obtained in the theme of AP and the intentionalizing (Int) bias suggest some specificity in psychosis. The underlying construct of the CBQp could be specifically related to interpretation bias, not being associated with reasoning, judgement, or decision-making processes (18).

Catastrophising (Cat) and JTC biases predict delusional experiences not only in subjects with schizophrenia but also in healthy subjects. In subjects diagnosed with schizophrenia, the Cat bias was the best predictor of the total severity index of delusions (measured by the PSYRATS), while the cognitive dimension of these delusions was specifically related to JTC (44).

Furthermore, Daalman et al. (45) compared clinical and healthy voice-hearers with controls, finding that most cognitive biases prevalent in clinical voice-hearers, particularly with threatening event themes, were absent in healthy voice-hearers, except for emotional reasoning which may be specifically related to the vulnerability to develop auditory verbal hallucinations (45).

Another recent study evaluated the impact of metacognitive training (MCT) on cognitive biases in people diagnosed with schizophrenia, finding improvements in the Cat, emotional reasoning (ER), and JTC biases, with an important impact on the CBQp total score (46). These results suggest that one of the first objectives of metacognitive training, to reduce cognitive biases (12), was reached in this sample of chronic patients (46). The results of a recent study with an MCT and psychoeducation intervention in recent-onset psychosis imply the usefulness of the CBQp to detect improvements in cognitive biases (47).

Similarly, another study investigated the relationship between cognitive biases and the cognitive and emotional dimensions of delusions in patients with schizophrenia spectrum disorders, controlling confounding variables such as hallucinations (48). The results show that the JTC bias was associated with both the delusion conviction and the associated emotional discomfort. Only the emotional discomfort associated with auditory hallucinations was related to dichotomous thinking (DT) and Int biases. These results are consistent with previous results that found that JTC, measured by the CBQp, was related not only to clinical delusions but also to a non-clinical propensity to delusions (44).

All of these data support the idea that JTC may be relevant throughout the different stages of delusion formation (32, 33) and could be a vulnerability–trait factor that could increase the risk of developing delusional experiences (44, 49, 50).

This study translated to Spanish the Cognitive Biases Questionnaire for Psychosis. Our objectives were to obtain the psychometric properties of the Spanish version of the CBQp, specifically to (1) analyze the factorial structure of this questionnaire and obtain the descriptive statistics of each dimension for patients with psychosis and controls, (3) obtain the reliability for internal consistency for each scale, (4) relate cognitive biases with a widely used scale in the field of delusion cognitive therapies for assessing metacognition, specifically Beck's Cognitive Insight Scale (1), and (5) associate cognitive biases with delusional experiences evaluated with the Delusions Inventory (PDI) (2).

## Methodology

### Participants and Procedures

This study had a cross-sectional design with a comparison group based on cases and controls matched for sex and age.

The validation of the CBQp was carried out in several stages. Authorization for the Spanish adaptation of the CBQp (18) was obtained from the authors (Peters E, personal communication, 2013). The linguistic and cultural adaptation of the scale was carried out using the methodology of direct and inverse translation (translation–back-translation) (51). To test the scale structure of the Spanish version of the CBQp, the questionnaire was administered to a sample of patients. The patient sample consisted of 171 subjects with psychosis, of whom 103 (60.23%) were men. The participants were outpatients (58%) and inpatients (42%). They were recruited from three main sites: Hospital Universitari Institut Pere Mata (Reus), Parc Tauli Hospital Universitari (Sabadell), and Parc Sanitari Sant Joan de Déu (Barcelona). Regarding the diagnoses, 84 (48.8%) participants were diagnosed with schizophrenia, 39 (22.9%) had an unspecified psychotic disorder, 28 (16.5%) had schizoaffective disorder, seven (4.1%) had schizophreniform disorder, six (3.5%) had bipolar disorder, four (2.4%) had brief psychotic disorder, and three (1.8%) had delusional disorder. There was no significant difference in age between male (*M* = 32.44, SD = 10.82) and female participants (*M* = 32.88, SD = 11.43) [*t* (169) = 0.25, *p* = 0.797].

Subsequently, to establish comparisons, the CBQp was administered to a group of patients and healthy subjects. To ensure that the group of patients and healthy subjects were matched by sex and age, a subsample of 157 patients, of whom 95 (60.5%) were men, was compared with the group of controls with 30 participants. A control group of 30 voluntary participants, of whom 17 (56.7%) were men, who had no diagnosed psychiatric disorder, was recruited from the community (Tarragona, Cataluña). No significant differences were found in terms of age between men (*M* = 31.71, SD = 6.70) and women (*M* = 32.77, SD = 7.65; *p* = 0.405). No significant differences were found in terms of sex between the two groups (χ^2^ = 0.155; *p* = 0.694). Information on the participants is reported in Table 1.

**Table 1:** Demographic information for the samples.

		**Control vs. Psychosis**			**Psychosis BCIS**	
**Groups**	**Psychosis** **CFA**	**Controls**	**Psychosis**	**Psychosis** **PDI**	**Low CI**	**High CI**
*N*	171	30	157	50	45	43
Gender (n) (%)	103M (60.2%)	17M (56.7%)	97M (39.5%)	30M (60%)	23M (51.1%)	31M (73.8%)
	68F (39.8%)	13F (43.3%)	62F (60.5%)	20F (40%)	22F (48.9%)	11F (26.2%)
		*p =* 0.694			*p =* 0.029	
Age in years (mean, SD)		32.17 (7.02) 33.01 (11.32)				
All		*p =* 0.594				
M	32.44 (10.82)	31.71 (6.70)	33.04 (10.97)	37.07 (10.15)	32.28 (11.51)	32.71 (11.29)
F	32.88 (11.43)	32.77 (7.65)	32.95 (11.92)	37.30 (12.44)	36.02 (12.59)	30.18 (12.64)
	*P =* 0.445	*p =* 0.405	*p =* 0.961	*p =* 0.942	*p =* 0.152	*p =* 0.540

*M, Male; F, Female; CFA, Confirmatory Factor Analysis; PDI, Peters Delusion Inventory; BCIS, Beck Cognitive Insight Scale; CI, Cognitive Insight*.

**Figure 1:**
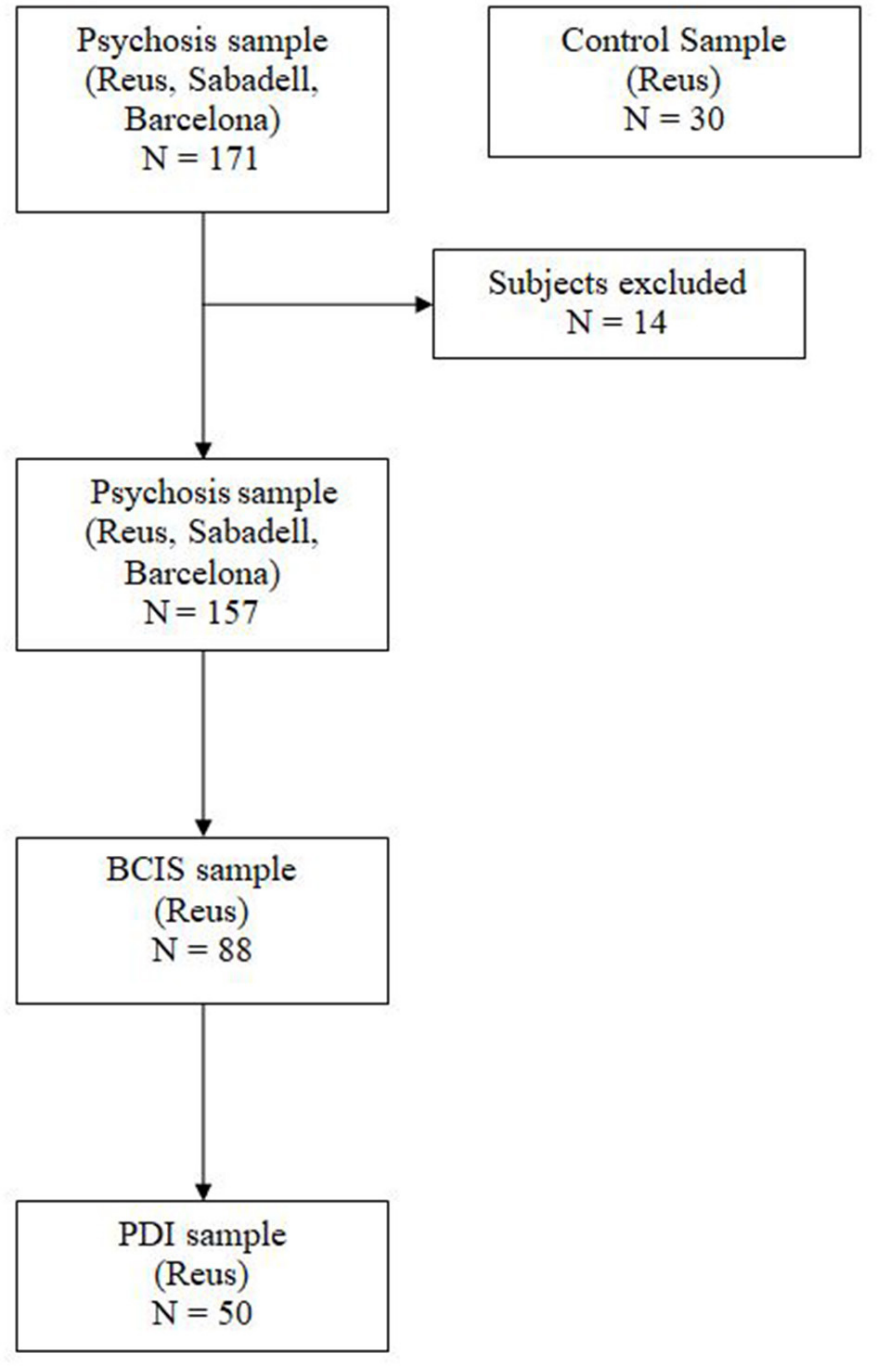
Sample selection.

The CBQp was administered together with the Beck Cognitive Insight Scale (BCIS) (1) and the Peters Delusions Inventory (2) to the Institut Pere Mata patients' group. BCIS was administered to both outpatients and inpatients, while PDI was only assessed in the inpatient sample (Figure 1).

All procedures were in accordance with the Declaration of Helsinki. Ethical approval was obtained from the local ethics committee (CEIm IISPV, www.iispv.cat). All the subjects consented to participate in the study and signed an informed consent form after a complete explanation of all procedures.

### Instruments

The CBQp (18) has a self-applied format with 30 descriptions of everyday situations, 15 on AP and 15 on TE, in which the subject must choose between three options that best describe how he or she would think about that situation. Each group of statements covers five cognitive biases: Int, Cat, DT, JTC, and ER. There are three statements per bias for each topic. Each vignette includes a forced choice on a three-point scale (1 = absence of bias, 2 = presence of bias with some qualification, 3 = presence of bias). The subject must imagine himself or herself in each situation and choose one of the three possible answers. Cronbach's alpha of the total CBQp was 0.89. The test–retest was 0.94 for the psychosis group and 0.70 for the healthy controls (18).

The BCIS (1) is a 15-item self-registration measure that assesses how patients evaluate their own judgement. It consists of two dimensions: self-reflectiveness (R) (nine items) and self-certainty (C) (six items). A composite index of cognitive insight is obtained as reflectiveness–certainty (IC = R—C) (subtraction of self-certainty from self-reflectiveness). The Cronbach coefficients of the self-reflectiveness and self-certainty for patients were 0.68 and 0.60, respectively (1). The internal consistency for the Spanish version of the BCIS was 0.59 for self-reflectiveness and 0.62 for self-certainty. The intraclass correlation coefficients of test–retest reliability were 0.69 for self-reflectiveness, 0.72 for self-certainty, and 0.70 for the composite index (52). Given that both of these subscales are composed of <10 items, the levels of internal consistency of the BCIS were considered to be acceptable for research purposes (53, 54), even though both coefficients were less than the 0.70 value recommended by Nunnally (55).

The Peters Delusion Inventory (2), with 21 items, consists of four scales to assess the presence/absence of delusional symptoms and their degree of conviction, preoccupation, and distress. For PDI, the Cronbach coefficient was 0.82. The test–retest reliability for the PDI yes/no and conviction scales was 0.78, while that for the distress and preoccupation scales was 0.81 (2). The Spanish version of the PDI had a Cronbach coefficient of 0.75 (56).

### Statistical Analysis

The psychometric properties of the scale structure, reliability, and validity of the Spanish version of the CBQp were analyzed:

1. Confirmatory factor analysis (CFA) was used to evaluate three alternative models of the scale construct, *i.e*., a five-factor model hypothesizing that each factor represents a separate bias, a two-factor model in which each factor represents a theme (AP and TE), and, finally, a one-factor model in which a general thinking bias underlies the five types of cognitive biases. For the CFA, the sample size was estimated to be five participants per item (30 items), with a necessary sample of at least 150 participants (57, 58). We used the weighted least square mean and variance adjusted estimation method, which is a robust estimator that does not assume normality and is the best option for modeling categorical or ordered data (59). To estimate the goodness of fit of the CFA, the estimators root mean square error of approximation (RMSEA), CFI, Tucker–Lewis index (TLI), and standardized root mean residual square (SRMR) were used according to the criteria of Hu and Bentler (60). Akaike's information criteria (AIC) (61) and Bayesian information criteria (BIC) (62) were also used to select the best model according to information criteria.

2. Analysis of the internal consistency to test the reliability of the CBQp scale was performed by means of Cronbach's alpha and composite reliability.

3. Several scales had a non-normal distribution. Differences between two groups of patients, based on the highest and the lowest scores according to the mean on the BCIS, and between samples (psychotic and healthy groups) were analyzed for the CBQp scales through the Mann–Whitney *U*-test, which is robust to non-normality. Comparisons between groups in terms of sex and age were carried out by χ^2^ and Student's *t*-test, respectively. Spearman's rho correlations were used to evaluate the association between themes and biases of CBQp and the BCIS and PDI scales.

The CFA and reliability calculations were conducted with the lavaan package (63) running under R 3.6.0 software, and the mean differences and correlations were conducted with SPSS v.23.

## Results

### Scale Factor Structure

The statistics for the factorial model of the CBQp of one, two (TE and AP), and five factors (Int, Cat, DT, JTC, and ER) are shown in Table 2. Although somewhat better for the five-factor model (CFI = 0.952, TLI = 0.948, RMSEA = 0.029, SRMR = 0.093, AIC = 8846.898, and BIC = 9066.815), the adjustment indices were also good and similar in the one-factor (CFI = 0.947, TLI = 0.943, RMSEA = 0.031, SRMR = 0.096, AIC = 8852.954, and BIC=9041.454) and two-factor models (CFI = 0.947, TLI = 0.943, RMSEA = 0.031, SRMR = 0.096, AIC = 8853.346, and BIC = 9044.987). The factors had significant positive correlations (*r*_s_ = 0.66, *p* < 0.001) when analyzed as themes, with the association between biases in the range of 0.34–0.78 (*p* < 0.001). The item factor loadings in each of the three models are presented in Table 3. Items 9, 19, and 27 had a factor loading < 0.3.

**Table 2:** Goodness of Fit for the Confirmatory Factor Analysis (CFA) (Psychosis group) (*N* = 171).

**CBQp**	**CFI**	**TLI**	**RMSEA**	**SRMR**	AIC^*^	BIC^*^
1-factor model	0.947	0.943	0.031 (0.015–0.042)	0.096	8852.954	9041.454
2-factor model	0.947	0.943	0.031 (0.015–0.042)	0.096	8853.346	9044.987
5-factor model	0.952	0.948	0.029 (0.012–0.041)	0.093	8846.898	9066.815

*CFI, Robust comparative fit index; RMSEA, Root mean square error of approximation; SRMR, Standardized root mean square residual; TLI, Tucker-Lewis index; AIC, Akaike's Information Criteria; BIC, Bayesian Information Criteria*.

* *Because of the nature of both AIC and BIC, they were computed from maximum-likelihood estimations of the models*.

**Table 3:** Cognitive Biases Questionnaire for Psychosis (CBQp) factor loadings from the CFA.

	**2 Factors (Themes)**			**5 Factors (Biases)**				
	**1 Factor**	**1 (TE)**	**2 (AP)**	**1 (Int)**	**2 (Cat)**	**3 (DT)**	**4 (JTC)**	**5 (ER)**
CBQ1	0.434	0.436	–	0.439	–	–	–	–
CBQ2	0.432	–	0.436	–	0.426	–	–	–
CBQ3	0.660	–	0.667	0.669	–	–	–	–
CBQ4	0.588	0.593	–	–	0.578	–	–	–
CBQ5	0.328	0.331	–	–	–	0.386	–	–
CBQ6	0.578	–	0.584	–	–	–	0.575	–
CBQ7	0.590	0.593	–	–	0.578	–	–	–
CBQ8	0.737	–	0.744	–	–	–	–	0.790
CBQ9	0.261	0.264	–	–	–	–	0.265	–
CBQ10	0.469	–	0.473	–	0.463	–	–	–
CBQ11	0.536	0.540	–	–	–	0.621	–	–
CBQ12	0.711	0.717	–	–	0.696	–	–	–
CBQ13	0.526	0.529	–	–	–	–	–	0.564
CBQ14	0.513	–	0.517	–	–	0.585	–	–
CBQ15	0.564	0.568	–	–	–	0.648	–	–
CBQ16	0.570	–	0.575	–	–	–	–	0.612
CBQ17	0.436	–	0.442	–	–	–	0.433	–
CBQ18	0.726	0.731	–	–	–	–	0.723	–
CBQ19	0.262	0.263	–	–	–	–	–	0.285
CBQ20	0.648	–	0.654	0.654	–	–	–	–
CBQ21	0.479	–	0.482	–	–	–	0.479	–
CBQ22	0.580	0.584	–	0.587	–	–	–	–
CBQ23	0.450	–	0.453	0.455	–	–	–	–
CBQ24	0.794	0.800	–	–	–	–	–	0.852
CBQ25	0.802	–	0.810	–	0.786	–	–	–
CBQ26	0.447	–	0.450	–	–	–	–	0.480
CBQ27	0.212	–	0.213	–	–	0.246	–	–
CBQ28	0.443	0.446	–	0.448	–	–	–	–
CBQ29	0.571	0.576	–	–	–	–	0.571	–
CBQ30	0.525	–	0.529	–	–	0.598	–	–

*TE, threatening events; AP, anomalous perceptions; Int, intentionalising; Cat, catastrophising; DT, dichotomous thinking; JTC, jumping to conclusions; ER, emotional reasoning*.

### Reliability

Cronbach's alpha was 0.87 for the total scale (30 items), 0.76 for the AP scale (15 items), and 0.78 (15 items) for the TE scale. The composite reliability was 0.92 for the total scale and 0.86 for both the AP and TE scales.

### Validity

#### Comparisons Between Patient and Control Samples

The descriptive statistics for the group of patients and the controls are shown in Table 4. The Mann–Whitney *U*-test showed that the group with psychosis had a higher score than the control group in the total score of the CBQp (Mdn = 41/Mdn = 38, *U* = 1,705, *p* = 0.017), TE (Mdn = 21/Mdn = 20, *U* = 1,826, *p* = 0.051), AP (Mdn = 20/Mdn = 18, *U* = 1,725.5, *p* = 0.020), Int (Mdn = 7/Mdn = 7, *U* = 1,758, *p* = 0.024), DT (Mdn = 8/Mdn = 7, *U* = 1,655, *p* = 0.009), and ER (Mdn = 8/Mdn = 7, *U* = 1,638, *p* = 0.006). The TE theme (Mdn = 21/Mdn = 20, *U* = 1,826, *p* = 0.051) maintained a trend toward statistical significance.

**Table 4:** CBQp differences between patients with psychosis and controls.

	**Psychosis** **(*N =* 157)**		**Control** **(*N =* 30)**		***z***	***p***
**CBQp**	**Mdn**	**M (sd)**	**Mdn**	**M (sd)**		
Total score	41	43.17 (8.90)	38	38.90 (3.90)	−2.396	0.017
Threatening events (TE)	21	22.25 (4.96)	20	20.07 (2.11)	−1.954	0.051
Anomalous perceptions (AP)	20	20.92 (4.51)	18	18.83 (2.27)	−2.331	0.020
Intentionalising (Int)	7	7.82 (1.87)	7	6.93 (0.94)	−2.265	0.024
Catastrophising (Cat)	8	8.67 (2.26)	8	8 (1.46)	−1.195	0.232
Dichotomous thinking (DT)	8	8.50 (2.23)	7	7.33 (0.92)	−2.628	0.009
Jumping to conclusions (JTC)	9	9.79 (2.32)	9	9.23 (1.50)	−0.945	0.345
Emotional reasoning (ER)	8	8.39 (2.44)	7	7.17 (1.48)	−2.762	0.006

#### Comparison Between Groups Based on Cognitive Insight

The difference between the groups when the total sample was divided into a group with an equal or higher score than the mean and a group with a score below the mean on the Cognitive Insight Scale (*M* = 6.42) (BCIS) is shown in Table 5. The Mann–Whitney *U*-test showed that the group with lower insight had a higher score than the group with higher insight in the total score of the CBQp (Mdn = 44/Mdn = 39, *U* = 631.5, *p* = 0.005), TE (Mdn = 22/Mdn = 20, *U* = 664.5, *p* = 0.011), AP (Mdn = 20/Mdn = 19, *U* = 642, *p* = 0.006), Int (Mdn = 8/Mdn = 7, *U* = 685.5, *p* = 0.015), Cat (Mdn = 9/Mdn = 8, *U* = 653, *p* = 0.008), and JTC (Mdn = 10/Mdn = 9, *U* = 636, *p* = 0.005). There were no differences between the groups according to the level of cognitive insight in the DT (Mdn = 9/Mdn = 8, *U* = 777.5, *p* = 0.107) and ER (Mdn = 8/Mdn = 8, *U* = 778.5, *p* = 0.108) biases.

**Table 5:** CBQp scale differences between groups based on Cognitive Insight.

**CBQp**	Low Cognitive Insight^*^ **(*N =* 45)**		High Cognitive Insight^**^ **(*N =* 43)**				
	**Mdn**	**M (SD)**	**Mdn**	**M (SD)**	**Mann-Whitney U**	***z***	***p***
CBQ total	44	45.66 (10.51)	39	40 (6.07)	631.5	−2.80	0.005
Threatening events (TE)	22	23.42 (5.62)	20	20.55 (3.67)	664.5	−2.53	0.011
Anomalous perceptions (AP)	20	22.24 (5.36)	19	19.44 (3.12)	642	−2.73	0.006
Intentionalising (Int)	8	8.35 (2.42)	7	7.09 (1.28)	685.5	−2.43	0.015
Catastrophising (Cat)	9	9.33 (2.58)	8	7.97 (1.59)	653	−2.66	0.008
Dichotomous thinking (DT)	9	8.93 (2.52)	8	8.09 (1.77)	777.5	−1.61	0.107
Jumping to conclusions (JTC)	10	10.31 (2.66)	9	8.83 (1.67)	636	−2.80	0.005
Emotional reasoning (ER)	8	8.73 (2.43)	8	8 (2.32)	778.5	−1.60	0.108

* BCIS score ≥ 6.42;

** *BCIS score < 6.42*.

#### Correlations Between CBQp Scores and BCIS and PDI Scales

The significant correlations between the CBQp and the BCIS and PDI scales are shown in Table 6. A positive association was obtained between self-certainty and both themes, all the biases, and the total score of the CBQp: TE (*r*_s_ = 0.30, *p* < 0.01), AP (*r*_s_ = 0.34, *p* < 0.01), Int (*r*_s_ = 0.40, *p* < 0.001), Cat (*r*_s_ = 0.23, *p* < 0.05), DT (*r*_s_ = 0.22, *p* < 0.05), JTC (*r*_s_ = 0.21, *p* < 0.05), ER (*r*_s_ = 0.30, *p* < 0.01), and total score (*r*_s_ = 0.35, *p* < 0.01). The two themes and biases of the CBQp showed negative associations with the Cognitive Insight Scale, with the exception of the DT and ER biases: TE (*r*_s_ = −0.26, *p* < 0.05), AP (*r*_s_ = −0.26, *p* < 0.05), Int (*r*_s_ = −0.30, *p* < 0.01), Cat (*r*_s_ = −0.28, *p* < 0.01), and JTC (*r*_s_ = −0.22, *p* < 0.05). Similarly, the total CBQp score was negatively associated with cognitive insight (*r*_s_ = −0.28, *p* < 0.01). The self-reflectiveness scale of the BCIS only showed an association with the catastrophising bias of the CBQp (*r*_s_ = −0.25, *p* < 0.05). All the scales of the CBQp correlated positively with the scales of the PDI, with the exception of the Int bias. The highest correlations were obtained between the total score of the CBQp, AP, and Cat and the frequency of delusions, with the range of associations from 0.50 to 0.58 (*p* < 0.001).

**Table 6:** CBQp correlations with BCIS and PDI scales.

	**BCIS (*N =* 88)**			**PDI (*N =* 50)**			
**CBQp**	**Self-reflectiveness**	**Self-certainty**	**Cognitive** **insight**	**Total Yes/No**	**Distress**	**Preoccupation**	**Conviction**
Total score		0.35^**^	−0.28^**^	0.56^***^	0.55^***^	0.55^***^	0.58^***^
Threatening events (TE)		0.30^**^	−0.26^*^	0.53^***^	0.48^***^	0.52^***^	0.53^***^
Anomalous perceptions (AP)		0.34^**^	−0.26^*^	0.51^***^	0.56^***^	0.51^***^	0.55^***^
Intentionalising (Int)		0.40^***^	−0.30^**^				
Catastrophising (Cat)	−0.25^*^	0.23^*^	−0.28^**^	0.52^***^	0.50^***^	0.50^***^	0.57^***^
Dichotomous thinking (DT)		0.22^*^		0.35^*^	0.35^*^	0.35^*^	0.29^*^
Jumping to conclusions (JTC)		0.21^*^	−0.22^*^	0.47^**^	0.50^***^	0.45^**^	0.50^***^
Emotional reasoning (ER)		0.30^**^		0.42^**^	0.46^**^	0.47^**^	0.46^**^

*** p < 0.001;

** p < 0.01;

* *p < 0.05*.

## Discussion

The objective of this study was to validate the Spanish version of the CBQp questionnaire in a sample of patients with psychosis. For this, the factorial structure of the different models of the underlying construct in cognitive biases was analyzed. We aimed to obtain the reliability of the scale and the relationship of the biases with the BCIS for evaluating patients' metacognitive capacity and a widely used scale in the field of cognitive therapy for delusions. Finally, we analyzed the relationship between the biases and the PDI scale, an instrument to assess the degree of conviction, preoccupation, and distress produced by delusional symptoms.

Related to the factor structure of the CBQp, our results imply that the three factorial solutions had a good fit. In the study with the original version of the scale, the two- and five-factor models did not fit the data if independence was assumed in the factors (18). With related factors, the two-factor model best fit the underlying structure of the scale, suggesting that the scores of the themes could be used separately. In the Spanish version of the CBQp, we obtained a significant association between the themes and between the biases. Given the extremely high between-factor correlations in the two-factor and five-factor models and the relatively small differences in fit, the principle of parsimony leads us to choose the one-factor model as the best model explaining the data. A one-dimensional model of the scale's construct would be more parsimonious. Our results are consistent with those obtained in the validation study of the German version of the CBQp. This study showed that the one-factor model is the one with the best goodness of fit, also showing good fit for two- and five-factor models (64). As has been justified previously, the CBQp evaluates a general thinking style that underlies the cognitive biases previously recorded by Beck, with some variations depending on the type of situation (18). Thus, the different biases seem to represent a general tendency to process information in a distorted and alarming way.

The reliability (internal consistency) for the CBQp (0.87) was satisfactory and similar to the English version (0.89) (18), Flemish version (0.86) (64), and Polish version (0.83) (46). On the other hand, the reliability of the *anomalous perceptions* and *threatening events* themes, with Cronbach α of 0.76 and 0.78, respectively, are in a “moderate–high” range of internal consistency (65). Additionally, elevated composite reliability points to the unidimensionality of a construct. In our case, the composite reliability for the full scale was 0.92, suggesting that a single construct underlies all of the items.

Analyzing descriptive statistics, the Spanish version of the CBQp showed similar scores to those obtained with the English version, although with some slightly lower values. Thus, for cognitive biases, our scores were approximately one point lower. This difference is greater in the total score of the scale, with an average of 47.3 in the English version (18) compared to 43.19 in ours. On the other hand, a study that was carried out with a spectrum of schizophrenic patients with or without delusions obtained scores of 60.91 and 58.98, respectively (44). In the *threatening events* and *anomalous perceptions* themes, two previous studies with psychotic patients found higher scores than our validation study (18, 44). These differences between studies could be due to the distinct composition of diagnoses and/or the severity, intensity, and frequency of delusions in the samples. In our study, the analysis was not controlled for the severity of the symptomatology, so in future studies it would be necessary to recruit homogeneous samples in terms of diagnoses and the type and intensity of symptoms to establish more precise comparisons.

When compared with the healthy subjects' group, the psychotic patients scored significantly higher in the CBQp total score, in AP theme, and in all the biases, except for Cat and JTC. These results are discordant with the differences found in the English CBQp version, where all biases were significantly higher in the group of subjects with psychosis (18). In our study, the absence of differences between the patients and the control group in the TE theme and in both the Cat and JTC biases could have two explanations. First, Beck's cognitive biases were initially developed to define a depression-associated thinking style, having a broad emotional component and not just a psychotic cognitive–perceptual component, which could justify the presence of these biases in a healthy population. Thus, it would have been interesting to evaluate depressive and anxiety mood in both samples to identify the subjects' tendency to a distorted thinking style due to emotional issues. In future studies, it would be necessary to control emotional variables for evaluating the specificity of these cognitive biases to psychosis. On the other hand, the number of subjects in our control sample was 30, which, although similar to the original validation study, could subtract representativeness from the comparison between patients and controls.

It should be noted that the only previous study that provides the exploration of differences between a sample of patients and controls related to CBQp scores is the validation study of Peters et al. (18). When they compared the psychosis group with a group of subjects with depression, no significant differences between the groups regarding the TE theme were obtained. Similarly, other non-intuitive results were obtained, as the cognitive biases of JTC and PD were superior in the depression group than in the psychosis group (18). These results could imply, as mentioned above, that CBQp could be assessing negative-emotionality content biases.

We would like to highlight that our validation study sample had a similar proportion of men and women. To date, few studies have analyzed the presence of sex differences concerning cognitive biases in psychosis, finding no significant differences (66, 67). However, de Vos et al. (66) suggest the possibility of a subtle effect of sex differences related to delusion-associated cognitive biases, which is necessary to carry out more extensive studies with more statistical power to detect it (65). Indeed sex differences in cognitive biases could be expected because of these biases' link to neuropsychological performance (19, 68, 69) and global functioning (70) and the previously found differences between men and women concerning these domains (71, 72). These differences have also been found in affective symptoms (72) and awareness and attribution of psychotic symptoms (73). Therefore, although our control sample's size is small, it could be representative, using similar male and female percentages to those used in the psychosis group.

To our knowledge, the association between cognitive biases (CBQp) and cognitive insight (BCIS) has not been studied. As an expected result, in our study, both the themes and the biases were related to the self-certainty dimension of the BCIS, which measures the degree of conviction in the “reality” of the delusion contents of patients, showing more self-certainty based on a greater presence of biases. Except for emotional reasoning and dichotomous thinking, all CBQp scales were associated with cognitive insight. Previous studies have also obtained results pointing out JTC's association with self-certainty (74), supporting the idea of overconfidence about one's own decisions in psychotic patients (1). On the other hand, other studies did not find any association between JTC and the BCIS scales (self-reflection, self-certainty, and cognitive insight index) (75). It should be noted that these studies used probabilistic tasks for assessing cognitive biases and not the CBQp (74, 75).

In our results, except for ER and DT, the CBQp scores were associated with cognitive insight, showing that it decreased with a greater presence of biases. Similarly, when the sample was divided into two groups concerning cognitive insight (high/low), the group with less insight showed a greater presence of cognitive biases (CBQp total score) as well as higher scores in both themes (TE and AP) and Int, Cat, and JTC biases of the questionnaire. Cognitive insight would respond to the objectivity, reflexivity, and openness of the subject to external feedback (1), suggesting a greater presence of cognitive biases and a lower personal ability to detect them, associated with low cognitive insight.

Moreover, the results supporting the improvement of cognitive biases and cognitive insight after MCT (46, 76) suggest that there may be common thought and information processing mechanisms for both mental phenomena. Specifically, several studies show JTC's decrease after MCT (17, 46, 47), sometimes by the simple fact of making the subjects aware of the presence of biases (13, 77, 78), which would go in the line of achieving adequate cognitive insight. Nevertheless, it would be necessary to continue expanding the evidence in this field, analyzing the role of cognitive biases in insight formation processes.

Regarding delusional symptoms, in our study, both themes and all biases, except intentionalizing, were associated with the PDI scales. These results suggest that not only a greater presence of delusional symptomatology but also increased concomitant conviction, concern, and distress are involved in cognitive biases. Jumping to conclusions has a similar association with both emotional and cognitive dimensions. Some authors suggest that it would be more related to delusions' conviction (4). Other authors (44, 48) also found an association between JTC and the cognitive dimension of delusions (conviction and worry). They obtained similar results concerning the emotional dimension (distress) (48), and this hypothesis was refuted in our study. Although correlations are similar, catastrophising would be associated first with the cognitive dimension of delusions (conviction) and second with the emotional dimension (distress). Similarly, the hypothesis of previous studies (79–81), which suggests that Cat is related to the distress caused by delusions, would be confirmed. In this sense, our findings delve into the idea that cognitive biases assessed by CBQp are related not only to the cognitive dimension of delusions but also to the emotional dimension. This could imply that deficits in the metacognitive components of information processing in psychosis should be assumed from the cognitive–emotional state of patients. However, some studies with CBQp found no relationship between cognitive biases and the emotional dimension of delusions (44), while others found no relationship with either dimension (48). Therefore, more evidence is needed.

In summary, the Spanish version of the CBQp has replicated the factorial model of the general thinking style construct of a tendency to process information in a distorted and alarming way. The questionnaire has excellent reliability. The themes and cognitive biases of the questionnaire have been associated with greater delusional symptomatology and, equally, with worse metacognition when assessed by cognitive insight. Therefore, the psychometric properties and validation of the Spanish version of the CBQp guarantee that this instrument can be used as an assessment of cognitive biases in the Spanish language. Given the importance of cognitive biases in cognitive and metacognitive therapies of psychosis (12), instruments such as the CBQp, designed in a format based on everyday situations, are very useful in the evaluation of these biases in the previous phase or in the maintenance of delusions.

This study has several limitations. First, the low factor weight of three questionnaire items implies a worse association of these items with their theoretical factor. However, this result could be a limitation from psychometry's point of view, but this does not necessarily mean that these items need to be excluded; these items could provide relevant information concerning the clinical construct, and because of this, they are useful in bias measurement. Second, the analysis did not control for the severity of the symptomatology. Subsequent studies should analyze the biases according to the type and intensity of the delusions. Third, given the emotional component in Beck's biases, it would have been appropriated to control for mood state with the CBQp. Finally, diagnostic heterogeneity and the limited availability of sociodemographic data could limit the generalization of the study's results. The healthy subjects' sample was selected from health professionals' environment. In future studies, it would be necessary to analyze the descriptive statistics of biases in a broader sample of the general population. This would ensure more representativeness of the results, being able to establish cutoff points regarding to the healthy population and facilitating data generalization.

## Data Availability Statement

The raw data supporting the conclusions of this article will be made available by the authors, without undue reservation.

## Ethics Statement

The studies involving human participants were reviewed and approved by Comitè Ètic d'Investigació Clínica Institut d'investigació Sanitària Pere Virgili (CEIm IISPV de Reus). The patients/participants provided their written informed consent to participate in this study.

## Author Contributions

LC, JL, SO, AG-Z, and AC designed the study and participated in the linguistic adaptation of the Spanish version of the CBQp. DG-P, GM, JL, and AG-Z performed the data analysis. JL, SO, AG-Z, VS-G, DP, JMC, JV, and EV participated in the interpretation of data. LC, JL, AC, MA, and AG-Z participated in the acquisition, analysis and interpretation of data. LC and AG-Z wrote the paper with input from all authors. All authors discussed the results and contributed to the final manuscript, revised the manuscript content, and approved the final version of the manuscript.

## Conflict of Interest

The authors declare that the research was conducted in the absence of any commercial or financial relationships that could be construed as a potential conflict of interest.
